# Risk and benefit for targeted therapy agents in paediatric phase I trials in oncology: a systematic review and meta-analysis protocol

**DOI:** 10.1136/bmjopen-2026-123756

**Published:** 2026-09-11

**Authors:** Joanna Ostrowska, Karolina Strzebonska, Marcin Waligora

**Affiliations:** 1 REMEDY, Research Ethics in Medicine Study Group, Department of Bioethics and Health Psychology, Jagiellonian University Medical College, Krakow, Poland

**Keywords:** Systematic Review, Meta-Analysis, Adverse events, ONCOLOGY, Clinical Trial, Child

## Abstract

**Abstract:**

**Introduction:**

Paediatric phase I oncology trials establish safe dosing and provide preliminary efficacy data for investigational anti-cancer agents in children. The aim of the proposed systematic review is to identify the risk and benefit ratio for targeted therapy agents in paediatric Phase I trials in oncology.

**Methods and analysis:**

We will search Embase and PubMed for paediatric Phase I cancer trials published from 2 March 2015 to 30 June 2026. Eligible studies are dose-escalation trials enrolling participants under 21 years with solid or haematological malignancies, testing targeted therapy agents. We will measure two co-primary outcomes: (1) risk—proportion of participants experiencing drug-related adverse events of grade 3, 4 or 5; and (2) benefit—objective response rate. Where data permit, proportions will be pooled using random-effects meta-analysis.

**Ethics and dissemination:**

This study will use publicly available data; therefore, ethics committee approval is not required. Results from the systematic review will be published in a peer-reviewed journal and presented at relevant conferences.

**PROSPERO registration number:**

CRD420261408673.

**STRENGTHS AND LIMITATIONS OF THIS STUDY**

- The planned systematic review was prospectively registered in PROSPERO, and this protocol adheres to PRISMA-P guidelines.
- The systematic review will be conducted and reported in accordance with PRISMA 2020 guidelines.
- Study selection and data extraction will be performed independently by two researchers, with arbitration by a third reviewer when required.
- High between-study heterogeneity is anticipated and will be addressed through pre-specified subgroup and sensitivity analyses.
- Risk of bias assessment will not be performed, as phase I clinical trials are primarily designed to evaluate safety and dose rather than comparative effectiveness.

## Introduction

 Paediatric phase I oncology trials establish the maximum tolerated dose (MTD) or the recommended Phase II dose (RP2D), characterise pharmacokinetic and pharmacodynamic profiles, and provide preliminary evidence of anti-tumour activity. They raise several distinct ethical challenges. First, minors under 16 cannot give consent for participation; proxy consent and age-appropriate child assent are required, which may compromise participants’ developing autonomy.^1 2^ Second, participants typically have advanced or refractory disease and have exhausted standard therapeutic options, which raises concerns about therapeutic misunderstanding.^3 4^ Third, the risk–benefit balance is inherently more uncertain than in later-phase studies: participants are exposed to investigational agents of uncertain toxicity in the pursuit of dose-finding rather than direct therapeutic benefit.^5^ Fourth, the rarity of paediatric cancers constrains sample sizes and limits statistical inference.^5^

The ethical acceptability of these trials is contested. Regulatory frameworks vary across countries in the risk level and degree of potential benefit required to justify including minors. The Declaration of Helsinki requires risks to be proportionate to anticipated benefits, but empirical data supporting this have been scarce.^6^

Current systematic reviews of Phase I oncology trials involving children do not specifically address targeted therapies—a class of drugs that has gained significant prominence in early-phase clinical trials involving children over the past decade. Previous systematic reviews have addressed the risk–benefit balance in Phase I oncology trials. Mackley *et al* explored toxicity and response in Phase I oncology trials of targeted single-agent anticancer therapies, including both adult and paediatric trials, and observed a 0% rate of treatment-related deaths and a 6.4% response rate.^7^ Cohen *et al* reported an overall dose-limiting toxicity rate of 12.1% and an objective response rate of 15.3%.^8^ Waligora *et al* analysed 170 paediatric Phase I trials of any therapy class published between 2004 and 2015 and reported a pooled objective response rate of 10.29% and a fatal (grade 5) adverse event rate of 2.09% across all agents.^5^ However, none of these reviews has focused specifically on the risk–benefit balance of targeted therapy agents in paediatric patients only, and all cover periods that predate the recent expansion of targeted therapy in paediatric early-phase oncology.

The ethical importance of accurately assessing this balance has been further highlighted by empirical research showing that a majority of members of the public consider the potential benefits of paediatric Phase I oncology trials to justify their risks.^9^

Therefore, a review specifically focused on targeted therapies is needed to serve as a basis for ethical evaluation, informed decision-making, the development of regulatory policies and the refinement of clinical trial designs.

### Methods and analysis

We will adapt methods from our previous analyses of risk and benefit in oncology clinical trials.^5 10–12^

### Registration

We developed this protocol following the Preferred Reporting Items for Systematic Reviews and Meta-Analysis Protocols (PRISMA-P),^13^ and the systematic review will adhere to PRISMA 2020 guidelines.^14^ The completed checklist for this protocol can be found in online supplemental file 1). We registered the systematic review in PROSPERO (the International Prospective Register of Systematic Reviews) before commencing the searches (PROSPERO ID: CRD420261408673).^15^

### Data source and search strategy

We will conduct a systematic search of PubMed and Embase for relevant articles and abstracts published between 2 March 2015 and 30 June 2026, using strategies that include keywords and suggested MeSH and Emtree terms, their synonyms, and closely related words. Searches will not be restricted by language. A draft of the search strategy for Embase and PubMed can be found in online supplemental file 2 (Search Strategy). Beyond these bibliographic databases, we will search the ClinicalTrials.gov registry to identify registered clinical trials with posted results.

In addition, we will hand-search the reference lists of all included studies and of recent systematic reviews on related topics to identify additional eligible trials that may not have been captured by the database search.

### Eligibility criteria

#### Study design

Paediatric phase I studies in oncology, defined as ‘small sample-size, generally non-randomised, dose-escalation studies that define the recommended dose for subsequent study of a new drug in each schedule tested’.^16^ We will also consider phase I/II studies if phase I results are reported separately. Both completed and terminated trials will be included to limit potential publication or reporting bias associated with restricting eligibility to completed studies.

We will exclude conference abstracts, study protocols, observational studies, retrospective studies, case reports, letters, editorials, and narrative reviews.

#### Participants

We will include (1) studies involving paediatric populations; (2) mixed cohort studies in which more than 50% of participants are younger than 21 years; (3) studies explicitly described as paediatric; and (4) studies in which results are provided separately for paediatric patients. We will include all types of cancer—both solid tumours and haematological malignancies of any type and stage. Studies including only patients with benign tumours or non-cancerous diseases and studies enrolling exclusively adult participants with no separately extractable paediatric data will be excluded.

### Interventions

We will include Phase I studies in which targeted therapy was the only systemic treatment. Studies combining targeted therapy with other modalities (eg, chemotherapy, chemoradiotherapy or adjuvant therapies) will also be included if results for targeted therapy are reported separately.

We define targeted therapy (monoclonal antibodies or small molecules or antibody drug conjugates) in accordance with definitions provided by D.D. Karp *et al* in the *Handbook of Targeted Cancer Therapy and Immunotherapy*^17^ and the National Cancer Institute.^18^ We defined an agent as targeted therapy if its primary mechanism of action involves selective binding to a molecular target expressed on tumour cells or in the tumour microenvironment that is directly required for tumour growth, survival, or progression. Agents whose primary mechanism relies on activating or modulating the host immune response to recognise and destroy tumour cells (eg, immune checkpoint inhibitors, CAR-T cells, non-specific immunostimulants and cancer vaccines) are classified as immunotherapy and excluded. In ambiguous cases, classification will be determined in consultation with an experienced oncologist. We will exclude studies of therapies administered only topically or regionally, as well as studies on cytotoxic chemotherapy, immunotherapy, surgery or radiotherapy used alone. If targeted therapy results are not reported separately, studies combining targeted therapy with chemotherapy, immunotherapy, surgery or radiotherapy will also be excluded. Supportive care without anticancer agents and other treatments such as antiviral agents, non-specific immunotherapy (e.g. interferons, interleukins, cytokines, GM-CSF), cancer vaccines and oncolytic virus therapy will not be included.

We will additionally exclude studies of hormone therapies and non-pharmacological modalities (eg, radiotherapy, surgery, stem-cell therapy, or any of these alone), except for targeted therapy combined with surgery when Phase I targeted-therapy results are reported separately.

### Comparator

Not applicable (single-arm studies).

### Outcomes

We will include studies reporting benefit, measured as objective response rate and defined as the proportion of participants with partial and/or complete response as reported by authors of the included studies. Additional benefit outcomes will include progression-free survival and overall survival.

We will include studies reporting risk, measured as drug-related grade 3–5 adverse events according to the Common Terminology Criteria for Adverse Events (CTCAE) version 6.0 (and earlier versions).^19^

Additional outcomes will include number of patients with dose-limiting toxicities (DLTs) and whether a maximum tolerated dose (MTD) or recommended phase II dose (RP2D) was established from the trial.

Studies reporting either Adverse Events or Objective Response Rates (ORR) will be eligible for inclusion. Studies reporting neither primary outcome will be excluded. To compare risk and benefits, we will analyse a cohort of studies where both outcomes will be reported.

### Study selection

We will export all identified records to EndNote software for management and documentation purposes. We will remove duplicates via automated and manual methods. Two reviewers will independently screen titles and abstracts, followed by full-text screening against the eligibility criteria. Disagreements will be resolved by discussion; any unresolved cases will be adjudicated by a third reviewer.

### Data extraction

The data extraction form will be piloted before use. Two reviewers will independently extract data on study characteristics (eg, funding, phase, location, study status), population (eg, number of enrolled and eligible participants, type of malignancy), intervention eg, number of drugs, agent name/s, outcomes (eg, objective responses, drug-related adverse events). Discrepancies will be resolved by discussion or, if needed, by a third reviewer.

In the case of multiple publications for the same study, the results from the full publication and/or the most recent version will be used in the extraction. If the NCT number is provided, additional information on the trial description, results and publications will be searched and extracted from ClinicalTrials.gov.

### Missing data

For studies with incomplete outcome reporting (eg, ORR reported only for a single cohort in a multi-cohort study), we will search ClinicalTrials.gov for corresponding trial records and posted results. Data from trial registry records may be used to supplement information missing from published reports. If complete outcome data remain unavailable after review of both sources, the study will be included in the qualitative synthesis but excluded from quantitative analyses requiring those data.

### Risk of bias (quality) assessment

Not applicable.

Phase I clinical trials primarily aim to assess safety, tolerability and dose-ranging rather than comparative efficacy, so traditional risk-of-bias assessments designed for evaluating internal validity of comparative outcomes are not applicable.

Similarly, GRADE assessment will not be performed, as it is designed to evaluate the certainty of evidence for comparative effectiveness outcomes, which are not the primary focus of phase I studies.

### Data synthesis

For the benefit outcome, if sufficient data are available, pooled response rates within each stratum will be estimated using meta-analysis with random-effects models and the restricted maximum likelihood (REML) estimator to account for between-study heterogeneity. For the risk outcome, we will pool the proportion of patients experiencing at least one drug-related grade 3–4 or grade 5 adverse event using the same random-effects framework. Heterogeneity will be assessed using the I² statistic and 95% prediction intervals. Statistical analyses will be performed in R using the metafor package. If feasible, the average number of treatment-related adverse events per patient will be estimated using Poisson regression.

### Publication bias

Publication bias will be assessed by visual inspection of funnel plots for meta-analyses including more than 10 studies. In addition, Egger’s regression test will be performed to statistically assess funnel plot asymmetry.

### Analysis of subgroups or subsets

If enough studies are included, the differences in the average objective response rate and average number of drug-related grade 3, 4 and 5 adverse events between various categories (eg, number of investigational drugs, type of malignancy or number of enrolled patients) will be investigated using meta-regression and Poisson regression analysis, respectively.

Planned subgroup analyses will examine: (i) type of malignancy (solid tumours vs haematological malignancies); (ii) therapeutic subclass (monoclonal antibodies vs small-molecule inhibitors vs antibody–drug conjugates); (iii) number of investigational drugs per study (single-agent vs combination); (iv) sample size (small vs large studies, eg, ≤ 100 vs > 100 participants); (v) publication year categorised in 3 year windows (2015–2017, 2018–2020, 2021–2023, 2024–2026) to assess temporal trends; and (vi) funding source (industry-sponsored vs non-industry-sponsored).

Planned sensitivity analyses will include (i) restriction to studies of paediatric-only populations, excluding mixed-cohort studies with paediatric subgroup analyses; (ii) restriction to studies reporting both co-primary outcomes; (iii) restriction to completed trials, excluding terminated trials; and (iv) restriction to studies with complete outcome data. Where feasible, we will also conduct exploratory comparisons of pooled estimates from this review with estimates from our previous systematic reviews of paediatric phase I trials of any therapy class (2004–2015) and paediatric phase II targeted therapy trials.

## Discussion

This protocol outlines a systematic review of risk and surrogate benefits in paediatric phase I oncology trials published between March 2015 and June 2026. This review builds on our previous study^5^ and extends a programmatic line of research from our group that has characterised the clinical development success rates and social value of paediatric phase I oncology trials,^10^ the risk–benefit balance of targeted therapy agents in paediatric phase II trials,^11^ and the timing of first-in-minor clinical trials of new cancer drugs.^12^ Together, these works will cover more than two decades of paediatric clinical trials, enabling a more precise evaluation of the risk-benefit ratio.

This is particularly timely given parallel regulatory frameworks that shape paediatric oncology drug development. In the European Union, the Pediatric Regulation (EC) No 1901/2006^20^ established mandatory paediatric investigation plans for new medicines. In the United States, the Paediatric Research Equity Act (PREA), supplemented by the Research to Accelerate Cures and Equity (RACE) for Children Act,^21^ authorised the FDA to require paediatric studies of new cancer drugs whose molecular targets are relevant to paediatric malignancies. Recent commentaries have analysed how these frameworks aim to promote paediatric studies of new cancer medicines.^22 23^ Such regulatory changes, together with the expansion of targeted therapies and new drug classes since 2015,^24^ are expected to increase the number and diversity of paediatric early-phase trials, making an updated synthesis of risk and benefit particularly relevant for ethical evaluation, informed decision-making, regulatory policy and trial design improvement.

### Interpretation of results

We will interpret pooled estimates of risk and benefit in a clinically and ethically meaningful way. First, we will describe the balance between the pooled objective response rate and the pooled grade 3–5 adverse event rate by tumour type and therapeutic subclass. This descriptive framework does not claim that a single numerical threshold defines an ethically acceptable balance; rather, it provides transparent empirical grounding for such judgements.^2 3^ Second, we will compare our estimates with those reported in previous systematic reviews of paediatric phase I oncology trials, particularly Waligora *et al*^5^ and Cohen *et al*,^8^ to assess whether risk and benefit have shifted with the increasing use of targeted therapies. Third, we will discuss our findings in the context of the Declaration of Helsinki’s requirement of proportionality^6^ and existing regulatory and ethical guidance on paediatric research.

The findings will inform the ethical evaluation of paediatric phase I trials and support future methodological and regulatory improvements in the field.

### Limitations

This review has several anticipated limitations. First, not all Phase I trials are registered in ClinicalTrials.gov, which may lead to publication and reporting bias despite our systematic search of the registry. Second, formal risk-of-bias assessment at the individual-study level will not be conducted, as standard tools are not designed for single-arm dose-escalation studies. Third, categorisation of certain agents as targeted therapy vs immunotherapy may be ambiguous; we address this through explicit classification criteria and reviewer consultation with an experienced oncologist.

### Ethics and dissemination

This review synthesises publicly available data and requires no ethics approval. All data used are publicly available.

### Patient and public involvement

None.

## Supplementary material

10.1136/bmjopen-2026-123756 online supplemental file 1

10.1136/bmjopen-2026-123756 online supplemental file 2
