# Supplementary material for: Risk and benefit for targeted therapy agents in paediatric phase I trials in oncology: a systematic review and meta-analysis protocol
Source: BMJ Open. 2026 Sep 11;16(9):e123756. doi: 10.1136/bmjopen-2026-123756 (PMC13583735; doi:10.1136/bmjopen-2026-123756)
Supplement: Supplementary file 1 [file bmjopen-16-9-s002.pdf]

Table 1. Search strategy

EMBASE
(('cancer' OR 'cancer'/exp OR cancer OR 'cancers' OR 'cancers'/exp OR cancers OR cancerous OR carcinom* OR 'neoplasm' OR 'neoplasm'/exp OR neoplasm OR neoplas* OR 'oncology' OR 'oncology'/exp OR oncology OR oncolog* OR 'tumor' OR 'tumor'/exp OR tumor OR 'tumors' OR 'tumors'/exp OR tumors OR 'tumour' OR 'tumour'/exp OR tumour OR 'tumours' OR 'tumours'/exp OR tumours OR 'solid tumor'/exp OR 'solid tumor' OR 'solid tumour'/exp OR 'solid tumour' OR 'solid tumours' OR 'malignancy' OR 'malignancy'/exp OR malignancy OR malignan* OR 'leukemia' OR 'leukemia'/exp OR leukemia OR 'leukaemia' OR 'leukaemia'/exp OR leukaemia OR leukemias OR leukaemias OR leucocythaemia OR leucocythemia OR leucocythaemias OR leucocythemias OR 'acute myeloid leukemia'/exp OR 'acute myeloid leukemia' OR 'acute myeloid leukaemia'/exp OR 'acute myeloid leukaemia' OR 'acute lymphoblastic leukemia'/exp OR 'acute lymphoblastic leukemia' OR 'acute lymphoblastic leukaemia'/exp OR 'acute lymphoblastic leukaemia' OR 'acute myelogenous leukemia'/exp OR 'acute myelogenous leukemia' OR 'acute myelogenous leukaemia'/exp OR 'acute myelogenous leukaemia' OR 'hematologic malignancy'/exp OR 'hematologic malignancy' OR 'haematologic malignancy'/exp OR 'haematologic malignancy' OR 'hematologic neoplasms'/exp OR 'hematologic neoplasms' OR 'haematologic neoplasms'/exp OR 'haematologic neoplasms' OR 'chronic myelogenous leukemia'/exp OR 'chronic myelogenous leukemia' OR 'chronic myelogenous leukaemia'/exp OR 'chronic myelogenous leukaemia' OR 'juvenile myelomonocytic leukemia'/exp OR 'juvenile myelomonocytic leukemia' OR 'juvenile myelomonocytic leukaemia'/exp OR 'juvenile myelomonocytic leukaemia' OR 'myelodysplastic syndrome'/exp OR 'myelodysplastic syndrome' OR 'myelodysplastic syndromes'/exp OR 'myelodysplastic syndromes' OR 'transient myeloproliferative disorder'/exp OR 'transient myeloproliferative disorder' OR 'myeloid malignancies' OR 'myeloid malignancy'/exp OR 'myeloid malignancy' OR 'lymphoma' OR 'lymphoma'/exp OR lymphoma OR 'hodgkin disease'/exp OR 'hodgkin disease' OR 'hodgkins disease'/exp OR 'hodgkins disease' OR 'sarcoma' OR 'sarcoma'/exp OR sarcoma OR 'osteosarcoma' OR 'osteosarcoma'/exp OR osteosarcoma OR 'ewing sarcoma'/exp OR 'ewing sarcoma' OR 'ewing tumor'/exp OR 'ewing tumor' OR 'ewing tumour'/exp OR 'ewing tumour' OR 'rhabdomyosarcoma' OR 'rhabdomyosarcoma'/exp OR rhabdomyosarcoma OR 'wilms tumour'/exp OR 'wilms tumour' OR 'wilms tumor'/exp OR 'wilms tumor' OR 'nephroblastoma' OR 'nephroblastoma'/exp OR nephroblastoma OR 'retinoblastoma' OR 'retinoblastoma'/exp OR retinoblastoma OR 'medulloblastoma' OR 'medulloblastoma'/exp OR medulloblastoma OR 'teratoma' OR 'teratoma'/exp OR teratoma OR 'germinoma' OR 'germinoma'/exp OR germinoma OR 'germ cell tumor'/exp OR 'germ cell tumor' OR 'germ cell tumour'/exp OR 'germ cell tumour' OR 'seminoma' OR 'seminoma'/exp OR seminoma OR 'gonadoblastoma' OR 'gonadoblastoma'/exp OR gonadoblastoma OR 'glioma' OR 'glioma'/exp OR glioma OR 'glioblastoma' OR 'glioblastoma'/exp OR glioblastoma OR 'astrocytoma' OR 'astrocytoma'/exp OR astrocytoma OR 'carcinoma' OR 'carcinoma'/exp OR carcinoma OR blastoma:ti,kw) AND ('phase 1 clinical trial':ti,kw,ab OR 'phase 1 trial':ti,kw,ab OR 'phase 1 study':ti,kw,ab OR 'phase 1 clinical study':ti,kw,ab OR 'phase i clinical trial':ti,kw,ab OR 'phase i clinical study':ti,kw,ab OR 'phase i study':ti,kw,ab OR 'phase i trial':ti,kw,ab OR 'phase i':ti,kw,ab OR 'phase 1/2':ti,kw,ab OR 'phase 1':ti,kw,ab OR 'phase i/ii':ti,kw,ab OR 'phase 1/2 clinical study':ti,kw,ab OR 'phase i/ii clinical study':ti,kw,ab OR 'phase 1/2 clinical trial':ti,kw,ab OR 'phase i/ii clinical trial':ti,kw,ab) AND (pediatrics:ti,kw,ab OR paediatrics:ti,kw,ab OR paediatric:ti,kw,ab OR pediatric:ti,kw,ab OR children:ti,kw,ab OR child*:ti,kw,ab OR child:ti,kw,ab OR childhood:ti,kw,ab OR adolescent:ti,kw,ab OR infant:ti,kw,ab OR

newborn:ti,kw,ab OR toddler:ti,kw,ab OR teen:ti,kw,ab OR 'young adult':ti,kw,ab OR teenager:ti,kw,ab OR preschooler:ti,kw,ab OR 'pre teen':ti,kw,ab) AND [2015-2026]/py) OR (('child'/exp OR 'pediatric'/exp) AND 'oncology'/exp AND 'phase 1 clinical trial'/exp)
**PUBMED**
(((pediatrics[Title/Abstract] OR paediatrics[Title/Abstract] OR paediatric[Title/Abstract] OR pediatric[Title/Abstract] OR children[Title/Abstract] OR child*[Title/Abstract] OR child[Title/Abstract] OR childhood[Title/Abstract] OR adolescent[Title/Abstract] OR infant[Title/Abstract] OR newborn[Title/Abstract] OR toddler[Title/Abstract] OR teen[Title/Abstract] OR young adult[Title/Abstract] OR teenager[Title/Abstract] OR preschooler[Title/Abstract] OR pre-teen[Title/Abstract]) AND ((phase 1 clinical trial[Title/Abstract]) OR (phase 1 trial[Title/Abstract]) OR (phase 1 study[Title/Abstract]) OR (phase 1 clinical study[Title/Abstract]) OR (phase i clinical trial[Title/Abstract]) OR (phase i trial[Title/Abstract]) OR (phase I clinical study[Title/Abstract]) OR (phase i study[Title/Abstract]) OR (phase)1[Title/Abstract] OR (phase i[Title/Abstract]) OR (phase i[Title/Abstract]))) AND (cancer OR cancers OR carcinoma OR cancerous OR carcinom* OR neoplasm OR neoplas* OR oncology OR oncolog* OR tumor OR tumors OR tumour OR tumours OR (solid tumor) OR (solid tumours) OR solid tumour OR solid tumours OR malignancy OR malignan* OR leukemia OR leukaemia OR leukemias OR leukaemias OR leucocythaemia OR leucocythaemia OR leucocythaemia OR leucocythaemia OR (acute myeloid leukemia) OR (acute myeloid leukaemia) OR (acute lymphoblastic leukemia) OR (acute lymphoblastic leukaemia) OR (acute myelogenous leukemia) OR (acute myelogenous leukaemia) OR (hematologic malignancy) OR (haematologic malignancy) OR (hematologic neoplasms) OR (hematologic neoplasms) OR (chronic myelogenous leukemia) OR (chronic myelogenous leukaemia) OR (juvenile myelomonocytic leukemia) OR (juvenile myelomonocytic leukaemia) OR (myelodysplastic syndrome) OR (myelodysplastic syndromes) OR (transient myeloproliferative disorder) OR (myeloid malignancies) OR (myeloid malignancy) OR lymphoma OR (hodgkin disease) OR (hodgkins disease) OR sarcoma OR osteosarcoma OR (ewing sarcoma) OR (ewing tumor) OR (ewing tumour) OR rhabdomyosarcoma OR (wilms tumour) OR (wilms tumor) OR nephroblastoma OR retinoblastoma OR medulloblastoma OR teratoma OR germinoma OR (germ cell tumor) OR (germ cell tumour) OR dysgerminoma OR seminoma OR gonadoblastoma OR glioma OR glioblastoma OR astrocytoma OR carcinoma OR blastoma)) OR (((((child[MeSH Terms]) AND ("clinical trials as topic"[MeSH Terms])) AND (humans[MeSH Terms])) AND (medical oncology[MeSH Terms])) AND (pediatrics[MeSH Terms]))
