# Supplementary material for: Risk and benefit for targeted therapy agents in paediatric phase I trials in oncology: a systematic review and meta-analysis protocol
Source: BMJ Open. 2026 Sep 11;16(9):e123756. doi: 10.1136/bmjopen-2026-123756 (PMC13583735; doi:10.1136/bmjopen-2026-123756)
Supplement: Supplementary file 2 [file bmjopen-2026-123756.reviewer_comments.pdf]

PEER REVIEW HISTORY

BMJ Open publishes all reviews undertaken for accepted manuscripts. Reviewers are asked to complete a checklist review form and are provided with free text boxes to elaborate on their assessment. These free text comments are reproduced below.

ARTICLE DETAILS

Title (Provisional)

Risk and Benefit for Targeted Therapy Agents in Pediatric Phase I Trials in Oncology: A Systematic Review and Meta-Analysis Protocol

Authors

Ostrowska, Joanna; Strzebonska, Karolina; Waligora, Marcin

VERSION 1 - REVIEW

**Reviewer**	**1**
**Name**	**Iskander, Renata**
**Affiliation**	**McGill University**
**Date**	**11-Jul-2026**
**COI**	**None**

Summary

The authors are planning to conduct an important study to compare risk (drug-related grade 3/4/5 adverse events) and benefit (objective response rate) in pediatric patients with cancer treated with target therapies in Phase 1 trials. They are planning a systematic review and meta-analysis using literature found in Embase, PubMed, and ClinicalTrials.gov. They will also look at other outcomes, such as progression-free survival and overall survival, where available. The results from this review will be relevant to patients, clinicians, trialists, IRBs, and policymakers. I applaud the study's methodological rigour. In my review, I hope to share some questions to consider, helpful suggestions, and minor comments to help improve the study protocol for publication as well as the study conduct. Overall, the protocol follows the PRISMA-P reporting guideline, was registered in PROSPERO, and is clear and well-written. I look forward to reading the final study.

Comments

1. Introduction: You write that these trials "raise ethical challenges" but then only list one challenge. More challenges are mentioned in your PROSPERO registration. I suggest expanding more here on what makes pediatric Phase 1 cancer trials challenging.

2. Introduction: Include a few examples of what other systematic reviews have found regarding the balance between risk and benefit in pediatric oncology trials.

3. Methods: Overall, your methods are well-written and easy to follow. However, I think the format can be improved by dissolving the Exclusion Criteria subsection and incorporating those points into your PICO subsections. In your PICO subsections, you already discuss some exclusion criteria, so a separate exclusion criteria subsection is not necessary.

4. Methods: Please specify in the inclusion criteria whether you are including only completed trials or if terminated trials will also be included. I think both should be included to limit biases.

5. Methods - Interventions: Please specify how you will distinguish targeted therapy and immunotherapy (for some drugs, the categorization is difficult). You briefly mention how you define immunotherapy in the Exclusion Criteria section, but this could be explained further and moved to the Interventions subsection.

6. Methods - Outcomes: Will you include studies that have only one of the primary outcomes or do they have to report both drug-related AEs and ORRs? I assume the former, but please specify.

7. Methods - Data Synthesis: This subsection only mentions pooling response rates but not adverse events. Can you clarify here how both primaries will be analyzed, pooled, and presented?

8. Methods – Data Synthesis: How do you plan to test for publication bias? Unfortunately, not all Phase 1 trials are registered in ClinicalTrials.gov, which may lead to bias.

9. Methods – Analysis of subgroups and subsets: If there are enough studies to allow for pooling, are there any planned subgroup or sensitivity analyses that you did not mention in this subsection? In accordance with the PRISMA-P reporting guidelines, please include a little more detail on these planned analyses. Furthermore, how many mixed cohort studies do you expect? If there is a high number, I might suggest doing a sensitivity analysis removing these studies. Finally, it will be interesting to compare your results with results from your previous systematic reviews (i.e. 2004-2015 vs. 2015-2026, Phase 1 vs. Phase 2).

10. Discussion: How will you interpret the results? How do you plan to use your data to draw conclusions about whether risks outweigh benefits or benefits outweigh risks?

11. Discussion: “The review is limited by its reliance on published data and the heterogeneity between studies, both of which will be addressed through pre-specified subgroup analyses.” This relates to comment #9, but what are some of these pre-specified analyses?

12. Discussion: Are there any challenges you anticipate during the review? How will you manage missing data? Are there any limitations that should be acknowledged in the protocol?

VERSION 1 - AUTHOR RESPONSE

REVIEWER COMMENTS

Comment 1

Introduction: You write that these trials "raise ethical challenges" but then only list one challenge. More challenges are mentioned in your PROSPERO registration. I suggest expanding more here on what makes pediatric Phase 1 cancer trials challenging.

Response: Thank you for pointing this out. We expanded the Introduction section to include other ethical challenges associated with pediatric Phase 1 cancer trials. This section now states:

"First, minors under 16 cannot give consent for participation; proxy consent and age-appropriate child assent are required, which may compromise participant developing autonomy [1,2]. Second, participants typically have advanced or refractory disease and have exhausted standard therapeutic options, which raises concerns about therapeutic misunderstanding [3,4]. Third, the risk–benefit balance is inherently more uncertain than in later-phase studies: participants are exposed to investigational agents of uncertain toxicity in the pursuit of dose-finding rather than direct therapeutic benefit [5]. Fourth, the rarity of pediatric cancers constrains sample sizes and limits statistical inference [5]."

Comment 2

Introduction: Include a few examples of what other systematic reviews have found regarding the balance between risk and benefit in pediatric oncology trials.

Response: Thank you for this suggestion. We added findings from previous systematic reviews to the Introduction:

"Mackley et al. explored toxicity and response in Phase I oncology trials of targeted single-agent anticancer therapies, including both adult and pediatric trials, and observed a 0% rate of treatment-related deaths and a 6.4% response rate [7]. Cohen et al. reported an overall dose-limiting toxicity rate of 12.1% and an objective response rate of 15.3% [8]. Waligora et al. analysed 170 pediatric Phase I trials of any therapy class published between 2004 and 2015 and reported a pooled objective response rate of 10.29% and a fatal (grade 5) adverse event rate of 2.09% across all agents [5]."

Comment 3

Methods: Overall, your methods are well-written and easy to follow. However, I think the format can be improved by dissolving the Exclusion Criteria subsection and incorporating those points into your PICO subsections.

Response: We revised the manuscript accordingly. We removed the Exclusion criteria subsection, and integrated its content into the corresponding PICO subsections.

Comment 4

Methods: Please specify in the inclusion criteria whether you are including only completed trials or if terminated trials will also be included. I think both should be included to limit biases.

Response: Thank you for this suggestion. The Study design subsection now clarifies:

"Both completed and terminated trials will be included to limit potential publication or reporting bias associated with restricting eligibility to completed studies."

Comment 5

Methods - Interventions: Please specify how you will distinguish targeted therapy and immunotherapy (for some drugs, the categorization is difficult). You briefly mention how you define immunotherapy in the Exclusion Criteria section, but this could be explained further and moved to the Interventions subsection.

Response: We added detailed classification criteria to the Interventions subsection, drawing on definitions from Karp et al. [17] and the National Cancer Institute [18]:

"We defined an agent as targeted therapy if its primary mechanism of action involves selective binding to a molecular target expressed on tumor cells or in the tumor microenvironment that is directly required for tumor growth, survival, or progression. Agents whose primary mechanism relies on activating or modulating the host immune response to recognize and destroy tumor cells (e.g., immune checkpoint inhibitors, CAR-T cells, non-specific immunostimulants, cancer vaccines) are classified as immunotherapy and excluded. In ambiguous cases, classification will be determined in consultation with an experienced oncologist."

Comment 6

Methods - Outcomes: Will you include studies that have only one of the primary outcomes or do they have to report both drug-related AEs and ORRs? I assume the former, but please specify.

Response: Thank you for this remark. We clarified the Outcomes subsection:

"Studies reporting either AEs or ORRs will be eligible for inclusion. Studies reporting neither primary outcome will be excluded. To compare risk and benefits, we will analyze a cohort of studies where both outcomes will be reported."

Comment 7

Methods - Data Synthesis: This subsection only mentions pooling response rates but not adverse events. Can you clarify here how both primaries will be analyzed, pooled, and presented?

Response: We rewrote the Data synthesis subsection. The revised text now states:

"For the benefit outcome, if sufficient data are available, pooled response rates within each stratum will be estimated using meta-analysis with random-effects models and the restricted maximum likelihood (REML) estimator to account for between-study heterogeneity. For the risk outcome, we will pool the proportion of patients experiencing at least one drug-related grade 3–4 or grade 5 adverse event using the same random-effects framework. Heterogeneity will be assessed using the I^2 statistic and 95% prediction intervals. Statistical analyses will be performed in R using the metafor package. If feasible, the average number of treatment-related adverse events per patient will be estimated using Poisson regression."

Comment 8

Methods – Data Synthesis: How do you plan to test for publication bias? Unfortunately, not all Phase 1 trials are registered in ClinicalTrials.gov, which may lead to bias.

Response: Thank you for raising this important point. We added a dedicated statement on publication bias assessment to the Data synthesis subsection:

"Publication bias will be assessed by visual inspection of funnel plots for meta-analyses including more than 10 studies. In addition, Egger's regression test will be performed to statistically assess funnel plot asymmetry."

Comment 9

Methods – Analysis of subgroups and subsets: If there are enough studies to allow for pooling, are there any planned subgroup or sensitivity analyses that you did not mention in this subsection? In accordance with the PRISMA-P reporting guidelines, please include a little more detail on these planned analyses. Furthermore, how many mixed cohort studies do you expect? If there is a high number, I might suggest doing a sensitivity analysis removing these studies. Finally, it will be interesting to compare your results with results from your previous systematic reviews (i.e. 2004-2015 vs. 2015-2026, Phase 1 vs. Phase 2).

Response: Thank you for these detailed suggestions. We expanded the Analysis of subgroups or subsets subsection with pre-specified subgroup analyses (type of malignancy, therapeutic subclass, number of investigational drugs, sample size, publication year, funding source) and sensitivity analyses (including restriction to pediatric-only populations, as suggested by the Reviewer). We also added exploratory comparisons with our previous systematic reviews.

Comment 10

Discussion: How will you interpret the results? How do you plan to use your data to draw conclusions about whether risks outweigh benefits or benefits outweigh risks?

Response: We added a dedicated Interpretation of results subsection to the Discussion. We describe a three-part interpretive framework: (1) descriptive presentation of pooled risk and benefit estimates by tumor type and therapeutic subclass, without claiming that a single numerical threshold defines an ethically acceptable balance; (2) comparison with prior systematic reviews of targeted agents (Waligora et al.; Cohen et al.), to assess whether the balance has shifted with the increasing use of targeted therapies; and (3) contextualization of findings within the Declaration of Helsinki's requirement of proportionality and selected regulatory and ethical guidance on pediatric research.

Comment 11

Discussion: "The review is limited by its reliance on published data and the heterogeneity between studies, both of which will be addressed through pre-specified subgroup analyses." This relates to comment #9, but what are some of these pre-specified analyses?

Response: We removed the vague statement and replaced it with an explicit list of pre-specified subgroup and sensitivity analyses (see response to Comment 9). The Discussion now references the specific analyses detailed in the Methods and explains how each addresses the corresponding source of heterogeneity or bias.

Comment 12

Discussion: Are there any challenges you anticipate during the review? How will you manage missing data? Are there any limitations that should be acknowledged in the protocol?

Response: We added a dedicated Missing data subsection to the Methods and a Limitations subsection to the Discussion, addressing incomplete trial registration, the absence of formal risk-of-bias assessment, and ambiguous targeted-therapy classification.
